# Dissecting the role of the gut microbiota and diet on visceral fat mass accumulation

**DOI:** 10.1038/s41598-019-46193-w

**Published:** 2019-07-05

**Authors:** Caroline I. Le Roy, Ruth C. E. Bowyer, Juan E. Castillo-Fernandez, Tess Pallister, Cristina Menni, Claire J. Steves, Sarah E. Berry, Tim D. Spector, Jordana T. Bell

**Affiliations:** 1 The Department of Twin Research and Genetic Epidemiology, Kings College London, 3-4th Floor South Wing Block D, St Thomas’ Hospital, Westminster Bridge Road, SE1 7E London, UK; 2 Department of Nutritional Sciences, King’s College London, Franklin-Wilkins Building, 150 Stamford Street, London, SE1 9NH UK

**Keywords:** Microbiota, Microbiome

## Abstract

Both gut microbiota and diet have been shown to impact visceral fat mass (VFM), a major risk factor for cardiometabolic disease, but their relative contribution has not been well characterised. We aimed to estimate and separate the effect of gut microbiota composition from that of nutrient intake on VFM in 1760 older female twins. Through pairwise association analyses, we identified 93 operational taxonomic units (OTUs) and 10 nutrients independently linked to VFM (FDR < 5%). Conditional analyses revealed that the majority (87%) of the 93 VFM-associated OTUs remained significantly associated with VFM irrespective of nutrient intake correction. In contrast, we observed that the effect of fibre, magnesium, biotin and vitamin E on VFM was partially mediated by OTUs. Moreover, we estimated that OTUs were more accurate predictors of VFM than nutrients and accounted for a larger percentage of its variance. Our results suggest that while the role of certain nutrients on VFM appears to depend on gut microbiota composition, specific gut microbes may affect host adiposity regardless of dietary intake. The findings imply that the gut microbiota may have a greater contribution towards shaping host VFM than diet alone. Thus, microbial-based therapy should be prioritised for VFM reduction in overweight and obese subjects.

## Introduction

Diet and lifestyle habits have changed drastically over the past century, contributing to excessive weight gain with detrimental impacts on human health. At present, over 12.5% of the worldwide population is considered obese (body mass index (BMI) > 30) compared to 5% in 1975^1,2^. This sharp increase in rates of obesity poses a continuing global health challenge. However, BMI is an imprecise measure of obesity and risk of its associated clinical consequences because it does not differentiate between different types of fat or lean mass that both exert effects on human health. It is the accumulation of abdominal fat, and specifically deep visceral fat mass (VFM) in the abdominal cavity, that has the most detrimental consequences on health^3^. VFM accumulation is a major risk factor for cardiovascular and metabolic disease, and cancer^4^. Both increased VFM and BMI are in part triggered by poor diet, decreased energy expenditure and sedentary life style^5,6^ that can be partly quantified^7^.

Although the majority of the dietary components are directly absorbed in the upper digestive tract, some nutrients such as fibre remain undigested when they reach the large intestine. The human gut is home to trillions of bacteria forming a complex ecosystem mediating host metabolic homeostasis^8^. Undigested food and unabsorbed nutrients can be fermented by intestinal microbes, providing a key nutritional source. By influencing the level and variety of substrates available for gut bacteria to grow upon, diet can shape the composition of the gut microbiota^9^. Changing dietary habits and the corresponding diet-mediated shifts in the gut microbiota are considered to be essential contributors to the growing obesity pandemic^10^. In return, diet can also be used to beneficially affect the symbiont. Yet, dietary interventions - the first line of care in obesity management, display mitigated results and to date have been insufficient in constraining the obesity epidemic. This absence of conclusive results suggests that dietary interventions aiming to improve host metabolism might be improved by also targeting the gut microbiota. This is further suggested by the fact that the gut microbiota may also play a role in individualised response to diet^11^. For instance, Dao *et al*., demonstrated that baseline levels of *A. muciniphila*, which was previously associated with improved gut barrier function and correction of metabolic endotoxemia in obese individuals^12^, was a predictor of metabolic response to a calorie restricted diet^13^.

However, to date it has proven difficult to separate the effects of diet and gut microbiota on host weight and metabolism, as the two are closely linked. Several studies using animal models have shown that microbes alone can trigger obesity-associated phenotypes and that diet alone may not be necessary to observe such effects^14^. Yet, demonstrating such effects in humans remains challenging. Intervention studies in humans have shown that administering a single bacterial strain can trigger reduced weight gain without dietary intervention^15^. However, the evaluation of the concomitant effects of diet and overall gut microbiome on obesity has not yet been carried out in large-scale studies potentially due to limitations in sample size and study design^16^. A better characterisation of the relative contributions of diet and gut microbiota towards obesity is of strong value for gut-targeted treatment development. Understanding the contribution of the gut microbiome in diet induced host adiposity is key towards an improved management of the obesity pandemic. Therefore, there is a need to understand the extent to which diet and the gut microbiota affect host adiposity synergistically or independently.

Using data collected on research volunteers from the TwinsUK cohort, we previously observed that VFM is strongly associated with gut microbiota composition^17^ and diet^6^ independently. Furthermore, multiple studies have found that diet patterns strongly associate with the composition of the gut microbiota. Thus, diet and the gut microbiota may synergistically affect host VFM. In this study, we aimed to characterise the relative contribution and inter-dependence of nutrient and gut microbiota impacts on host VFM by exploring the association between VFM, gut microbiota composition and nutrient intakes in a cohort of 1760 elderly female twins. This was pursued by combining pair-wise association analysis with conditional and mediation analyses, as well as random forest classifiers. The results will contribute towards our understanding of the potential role of the gut microbiota in health management.

## Results

### Cohort description

We explored the impact of gut microbiota and diet on VFM in 1760 older female twins (Table 1). Gut microbiota associations with VFM were assessed using 512 operational taxonomic units (OTUs) generated by sequencing of the V4 region of the 16S rRNA gene. Daily nutrient intake was estimated from self-reported diet questionnaire (FFQ) recorded following the EPIC-Norfolk guidelines^18^, resulting in 44 independent variables (Supplementary Table 1).

**Table 1:** TwinsUK sample descriptive statistics.

	Average
Age	68.33 ± 9.76 (years)
Gender	Female: 100%
BMI	25.87 ± 4.37
VFM	531.99 ± 281.57 (g)
Kcal/day	1918.21 ± 479.99

### Nutrients and gut microbes are associated with VFM

We first assessed the pair-wise associations between OTUs and VFM, and nutrients and VFM separately using linear regression correcting for BMI, age and family structure. We then carried out pair-wise association tests between nutrients and OTUs, and contrasted the pair-wise association results.

**Figure 1:**
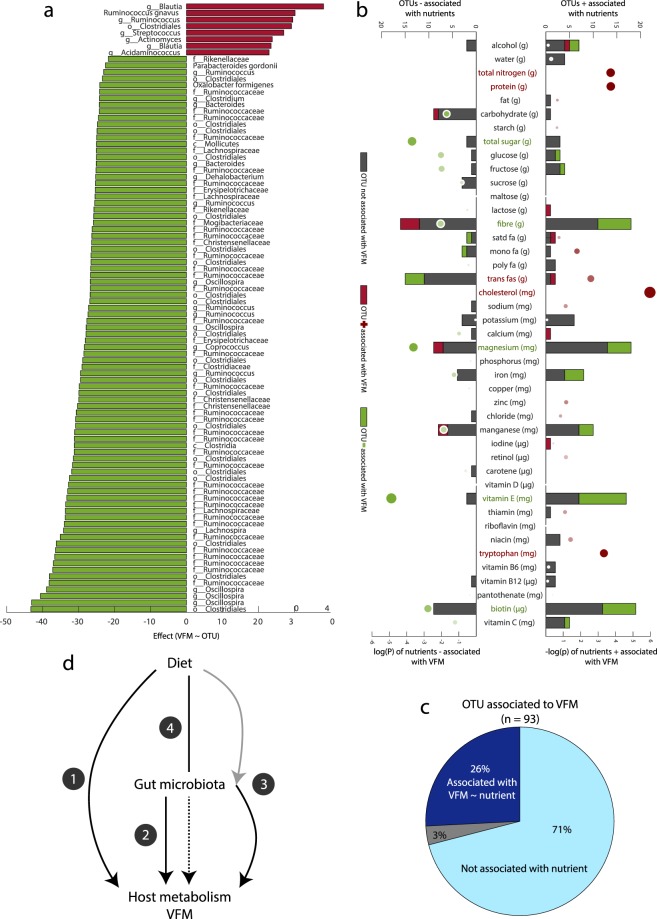
VFM is strongly associated with gut microbiota composition and nutrient intake. (**a**) OTUs significantly associated with VFM using OTU as predictor of VFM and correcting for age, energy intake (kcal), and family structure. Red bars represent positive associations and green bars show negatives associations. (**b**) Dietary component associations with VFM and OTUs. Bubble plot represents the –log transformed p-value with negative associations (left side) and positive associations (right side) resulting from the prediction of VFM by each food component independently, correcting for age, energy intake (kcal), and family structure. The colour intensity represents the effect size, red representing positive associations and green showing negatives associations. The bar chart represents the number of significant (FDR < 5%) OTUs negatively (right) or positively (left) associated with each food component. The bars are coloured in relation to significance and direction of the association of OTUs with VFM: grey - not associated, red - positively associated, green - negatively associated. (**c**) Proportion of VFM-associated OTUs associated with one VFM-associated food component (dark blue), a non-VFM-associated food component (grey) or no food (light blue). (**d**) Potential mechanisms of action of diet and microbiota effects on host metabolism or VFM deposition. Four pathways are shown: (1) diet directly impacts the host without GM mediation (i.e. nitrogen, protein, cholesterol and tryptophan); (2) gut microbes (OTUs) directly impact VFM independent of the effect of diet; (3) microbes impact VFM and these effects can be modulated by diet; (4) dietary impacts on VFM require specific gut microbial composition.

Pair-wise associations between OTUs and VFM revealed 93 OTUs significantly associated with VFM (FDR < 5%; Fig. 1a), where the majority displayed negative associations with VFM (n = 85; 91%). Most signals belonged to the Fimicutes phylum except for 6 Bacteroidetes, one Proteobacteria and one Tenericutes. Consistent with previous findings^19^ most Firmicutes OTUs were classified under the Rumminococcaceae family (41 OTUs) and the most represented genera was *Oscillospira* with 5 OTUs negatively associated with VFM, of which four presented the strongest associations with VFM. Only 8 OTUs were positively associated with VFM. All 8 OTUs but *Actinomyces* belonged to the Clostridiales order. Most positive associations were for OTUs classified within the *Blautia* genera. Among the 93 VFM-associated OTUs, 27 were also associated with at least one nutrient in the linear regression framework and surpassing FDR = 5%.

Pair-wise associations between nutrient intake (adjusted for total calorie intake) and VFM identified signals at ten of the 44 estimated nutrients, in linear regression analyses carried out as described in the above paragraph (P < FDR 5% Fig. 1b). Levels of nitrogen, protein, trans fatty acids, cholesterol and tryptophan were positively associated with VFM, while total sugar, fibre, magnesium, vitamin E and biotin displayed negative associations (Fig. 1b). We also evaluated the number of OTUs significantly associated with each nutrient independently to evaluate their potential as gut microbiota modulators (Fig. 1b). We then evaluated if the 10 nutrients significantly associated with VFM were also associated with OTUs using linear regression. Within the 10 VFM-associated nutrients five did not associate with any of the 582 OTUs, and these included cholesterol, protein, tryptophan and total nitrogen (a surrogate of protein intake). For the remaining six VFM-associated nutrients, the highest number of significant nutrient-OTU associations was observed for dietary fibre (34 associations), followed by biotin (28 associations) and magnesium (27 associations), and these were nutrients negatively associated with VFM. VFM-associated dietary components trans fatty acids, vitamin E and total sugar also presented some associations with gut microbiota composition (21, 19 and 6 respectively). For fibre, trans fat, magnesium, biotin and vitamin E, but not for total sugar, a proportion of the nutrient-associated OTUs were also associated with VFM. This last observation suggested that the effect of these nutrients on VFM might be partly mediated by gut microbiota modulation. Another 26 non-VFM-associated nutrients were also found to be significantly associated with at least one OTU, where manganese displayed the most significant associations with 19 OTUs (Fig. 1c). The number of OTUs significantly associated with the complete list of nutrients is available in Supplementary Table 2.

Thus, both gut microbiota composition and nutrient intake, independent of total energy intake, are strongly associated with VFM. As expected, we observed that a number of VFM-associated nutrients were also associated with VFM-associated OTUs and *vice versa* likely because the gut microbiota composition is modulated by diet.

### Nutrient intake does not have major effects on gut microbiota associations with VFM

We next carried out a series of conditional analyses to explore if nutrients and gut microbiota may influence host adiposity through four potential processes (Fig. 1d): (1) nutrients impact VFM directly without impacting or requiring gut microbiota mediation; (2) microbial taxa impact VFM directly without requiring the presence of nutrients; (3) nutrients impact the abundance of taxa that have a downstream effect on VFM; (4) nutrients are metabolised by gut bacteria and their by-products impact VFM.

**Figure 2:**
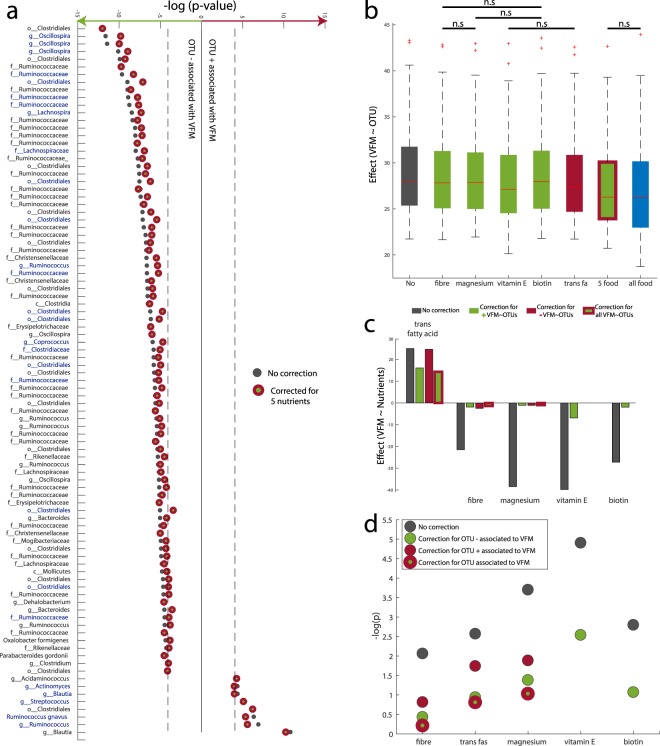
Dietary impacts on VFM depend on gut microbiota composition, but the opposite trend does not apply. (**a**) Significance of the association between VFM and VFM-associated OTUs after correction for 5 nutrients. (**b**) Variation in the absolute effect size of OTUs on VFM, depending on dietary component adjustment; all results are at FDR < 5% unless noted by n.s. (not significant). (**c**) Significance of the association between VFM and dietary components after correcting for VFM-associated OTUs relative abundance. (**d**) Significance of association between VFM and 93 OTUs represented as –log(P-value) while correcting for different dietary components.

To evaluate the contribution of nutrient intake towards the relationship between gut microbiota composition and VFM, we carried out a conditional analysis by linear regression assessing the associations between the 93 OTUs and VFM while correcting for nutrient intakes. The effect of nutrients on the association between VFM and OTUs was assessed by evaluating the number of OTUs that remained significantly associated with VFM (FDR 5%) after correction for nutrient intake, as well as the effect of the correction on the effect size of the association. To this end, we estimated the associations between VFM and the 93 VFM-associated OTUs while correcting for the five nutrients that were significantly associated with both VFM and OTUs (fibre, magnesium, trans fatty acids, vitamin E and biotin). The conditional analyses were carried by correcting for each nutrient separately, and then correcting for all 5 adiposity-associated nutrients in an additive model, and lastly a correction for all 44 nutrients was also applied. While in all cases the effect size of the association between VFM and OTUs was attenuated after correction for nutrient intake, the majority of these associations remained significant after correction for nutrient intake (Fig. 2a,b). Correction for all five nutrients together (fibre + magnesium + vitamin E + biotin + trans fatty acids) had the greatest impact on the reduction of the effect size. In total 81 (87%) of the 93 OTUs remained significantly associated with VFM after dietary correction, and the average effect size of the association was reduced by 5.8% after correction (Supplementary Tables 3 and 4). Most of the attenuated OTUs affected by the correction belonged to the Clostridiales order (n = 7). Surprisingly, the results from the correction for 44 nutrients were very similar to those from correction for the five nutrients alone, which could be due to the fact that the 5 nutrients may capture a large proportion of dietary variance. The greatest effect size reduction observed when correcting for single nutrients alone was obtained for vitamin E (89 of the 93 OTUs remained significant and the average effect size of association was reduced by 3.2%). Correction for vitamin E affected the association between VFM and OTUs classified under the *Bacteroides*, *Oxalobacter* and *Acidaminococus* genera and the Rikenellaceae family.

### Fibre, vitamin E, biotin and magnesium impacts on VFM depend of GM composition

**Figure 3:**
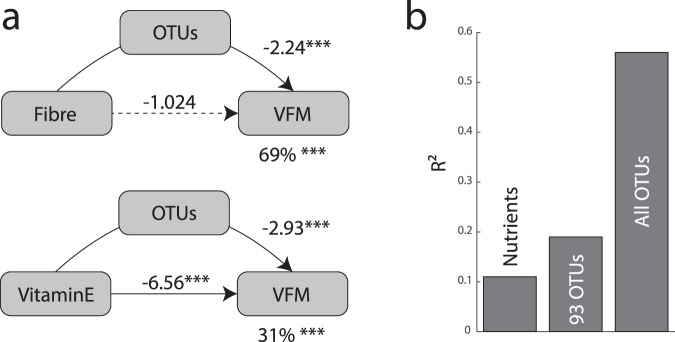
OTUs mediate the effects of nutrients on VFM. (**a**) OTU mediation of fibre and vitamin E on VFM. In each model the left box represents the causal variable (the nutrient), the top box is the mediator (OTUs, 1^st^ PC for the OTUs listed for each nutrient in Supplementary Table 6) and the right box is the response (VFM). The number by the top arrow represents the average causal mediation effect (ACME) and the one on the bottom arrow is the average direct effect (ADE). The number under the VFM box the percentage of mediation. *P < 0.05, **P < 0.01, ***P < 0.001. (**b**) Proportion of VFM variance attributed to OTUs and nutrients (R^2^) in a linear model.

We explored the role of the gut microbiota in mediating the association between nutrients and VFM. We carried out further conditional analyses now assessing the association between nutrients and VFM, correcting for adiposity-associated OTUs. This time, the effect of OTUs on the association between VFM and nutrients was assessed by evaluating if the nutrient-VFM association remained significant (p < 0.05) after correction for OTUs. In this section, we only considered correction by OTUs that were associated with both VFM and each nutrient independently (the list of OTUs used as covariate for each nutrient is available in Supplementary Table 5). This conditional analysis showed that correction for gut microbiota composition results in a major reduction in the strength of association between VFM and nutrient intakes (Fig. 2c and d). After correction, the associations between VFM and fibre, magnesium, biotin or trans fatty acids were no longer nominally significant (P > 0.05). Only the association between vitamin E and VFM remained significant after OTU correction. We further explored these effects by formal mediation analysis where OTUs were fitted as mediator of the effect of nutrients on VFM. We observed a full mediation effect of OTUs for fibre, biotin and trans fat where only the average causal mediation effect (ACME) model was significant (Fig. 3a and Supplementary Fig. 1). In total OTUs mediated 69% (P < 0.001), 43% (P < 0.001) and 56% (P = 0.02) of the effect of fibre, biotin and trans fat, respectively. On the other hand only partial mediation was observed for magnesium and vitamin E, where both the ACME and the average direct effect (ADE) models were significant. Nonetheless, in these two models OTU mediation was still estimated at 41% (P < 0.001) and 31% (P < 0.001) for magnesium and vitamin E, correspondingly.

### Gut microbiota composition explains more VFM variance than nutrients

To compare the relative variance in VFM accumulation that is captured by GM composition compared to nutrient intake, we evaluated the proportion of variance in VFM that is attributed to OTUs and nutrients in a sample subset of unrelated individuals (n = 982). The subsample of 982 unrelated individuals was selected by taking one twin per twin pair at random. We observed that OTUs capture a greater proportion of the VFM variance (R^2^ = 0.56 using all 512 OTUs and R^2^ = 0.19 using the 93 VFM-associated OTUs), which was nearly double that captured by all nutrients (R^2^ = 0.11; Fig. 3b).

We next assessed the predictive potential of GM composition and nutrient intake towards VFM accumulation. To this end, we applied a random forest classifier using multiple predictors of VFM accumulation^20^. The sample was split randomly between a training set (n = 700) to build the classifier, and a test set (n = 282) to evaluate model’s prediction potential. We initially assessed the predictive potential of the 44 nutrient intakes on VFM. Using the test set we estimate that VFM levels predicted by this model were relatively weakly correlated to observed VFM (r = 0.12; P = 0.046; RMSE = 0.11; Supplementary Fig. 2). In contrast, fitting the classifier with the 93 adiposity-associated OTUs alone resulted in predicted VFM levels that showed slightly greater correlation with observed VFM (r = 0.19, P = 0.0013, RMSE = 0.31). Lastly, we fit the RF classifier using the full set 93 OTUs and 44 nutrients jointly, and obtained the greatest correlation between predicted VFM and observed VFM (r = 0.23, P = 8.4 × 10^−5^; RMSE = 0.24).

## Discussion and Conclusion

The gut microbiota and nutrient intake can have synergistic impacts on host health. Yet, the contribution of each component is difficult to estimate in large-scale population studies. In this paper we tackled this issue by using pair-wise association and conditional analyses, along with machine learning approaches, to estimate and separate the effects of the gut microbiota and diet on host visceral fat mass (VFM) accumulation. We confirmed that gut microbiota composition and diet are both associated with VFM accumulation and that these two factors are closely linked, as over 70% of all nutrients were associated with at least one OTU. We then explored to extent to which the links between nutrients and OTUs may affect their association with VFM.

We first assessed the role of diet on the association between OTUs and VFM. We observed that OTU relative abundance, even after correction for nutrient intake, is strongly associated with VFM indicating that bacteria may not require the presence of nutrients to affect host phenotypes. The independent impact of the gut microbiota on host phenotypes has been extensively demonstrated in animal models^14,21^. In humans, faecal microbiota transplantation from lean donors has successfully improved insulin sensitivity in obese recipients^22,23^. Additionally, probiotic interventions in humans have also demonstrated the potential of administering a single microbial organism to affect host adiposity^16^. While our results together with the literature demonstrate that the presence of gut microbes regardless of diet is sufficient to alter host phenotypes, nutrients can still have an effect on this relationship. In our results nutrients reduced the average effect size of the association between OTUs and VFM. Hence, the gut microbiota may impact host VFM synergistically with nutrients.

We then explored the putative role of the gut microbiota on VFM-nutrients associations. Our results suggest that four nutrients: fibre, vitamin E, magnesium and biotin are most likely to require gut microbiota mediation to affect VFM accumulation. Fibre - the nutrient associated with the highest number of OTUs belonging to two well-known fibre degrading bacterial families: Ruminococaceae^24,25^ and Lachnospiraceae^26,27^ - was negatively associated with VFM. Gut microbiota composition appeared as a strong mediator of the fibre-VFM associations as we observed that 69% of the effect of fibre was mediated by OTUs. Fibres fall under the definition of prebiotics as they are non-digestible compounds that are degraded in the lower digestive track by intestinal microbes^28^. Both their ability to selectively enhance the growth of beneficial microbes as well as the by-products of their metabolism by gut bacteria are recognised to benefit host health^29,30^ and improve long term weight gain management^31^. Our results for fibre, consolidated by the existing literature, indicate that the conditional analysis used in this study can efficiently identify dietary nutrients requiring the presence of gut microbes to impact host VFM. Further confirmation was obtained with magnesium (Mg) - a nutrient negatively associated with the prevalence of cardio-metabolic disease in adults and children^32,33^ that may also be important for the ageing muscle^34^. The primary sources of Mg in the UK diet are vegetables, fruit and cereals and these are also high in fibre and non-nutritive bio-actives, which may in part explain the observed mediation by the gut microbiome^35^. Several studies have revealed that Mg absorption in the gut can be improved with fibre intake^36^, suggesting that the gut microbiota may play a potential role in its absorption. Additionally, Mg deficiency in mice has been associated with inflammatory response and shift in the gut microbiota composition^37,38^. While previous work does not show strong support for a synergistic effect of the gut microbiota with vitamin E and biotin on host metabolism, here we observed significant partial mediation of OTUs on the effect of these two nutrients on VFM (43% and 31% respectively). Nonetheless, approximately 30% of dietary vitamin E (α-tocopherol) is excreted in faeces^39^, thus, is in direct interaction with gut microbes. Vitamin E is an important antioxidant in the UK diet^39^, and is primarily derived from spreads, supplements and vegetables. It has been associated with lower risk of coronary heart disease^40^ and improved liver function in obese children^41^ due to its important role in lipid absorption. Despite the ability of vitamin E supplementation to improve colitis-associated syndromes^42^ and its negative association with Crohn’s disease^37^, the impact of vitamin E on the gut microbiota composition remains largely unexplored. Our results suggest that future studies should focus on the potential role of the gut microbiota in mediating the effect of vitamin E on host health. Biotin (vitamin B7) is exclusively synthetized by plants and gut bacteria, and has a role in the carboxylation of pyruvate to oxaloacetate and the initiation of fatty acids biosynthesis. The primary sources of biotin in the UK diet are supplements, eggs and semi-skimmed milk. However, little is known about its impact on the human gut microbiota and its potential effects on health. Surprisingly, we observed little or no association between OTUs and other B vitamins (B6 and B12).

On the contrary, mediation and conditional analyses conducted on trans fatty acids that were associated with both VFM and OTUs revealed that the observed effect of this nutrient on host adiposity was not likely to be mediated by the gut microbiota. In addition, our data also show that some nutrients appear to be directly associated with VFM without gut microbiota influences, and these included cholesterol, tryptophan, protein and total nitrogen. These results are surprising as dietary cholesterol absorption in the jejunum and duodenum is entirely dependent on bile acids^43^, and is one of the most studied example of host-gut microbiota metabolic cross talk^8,44^. Furthermore, tryptophan, protein and total nitrogen are markers of meat consumption. An effect of meat consumption on the gut microbiota has been reported in several studies^45^. Comparison of vegans and omnivores has revealed that while their metabolic signatures were drastically different, only subtle variations were observed in gut microbiota composition^46^. Interestingly, despite the absence of significant microbial variation between omnivores and vegans, there were key differences in their urine bacterial co-metabolite profiles^47^. This suggests that an omnivore diet may not alter the gut microbiota composition, but rather its metabolic activity. Thus, our study could be improved by looking at bacterial metabolic responses to diet which may be more informative in assessing the effect of certain nutrients on health, compared to gut microbiota itself^48,49^.

Overall, these results can help to understand the synergistic and individual actions of the gut microbiota and diet on the host in large population based cohorts. Characterising the interactions between host, gut microbiota and their environment is one of the main challenges of cross-sectional human microbiome studies. Thus, our approach can also be extended and applied to obtain insights into the role of the gut microbiota and other environmental factors on human health. Yet, our study also has a number limitations. First,  it depends on the assumption that the environment (here diet) exerts a causal effect on both the gut microbiota and the host, and that the gut microbiota causally affects the phenotype. In addition, mediation analysis does not allow us to account for several mediators at the same time. We therefore used microbiome data principal components (PCs) instead of individual OTUs to assess the mediation potential of the gut microbiota. This approach may not capture the full effect of individuals microbes as well as total gut microbiota on VFM-nutrient associations. Another limitation to our study is that the results are based on statistical associations analyses based on cross-sectional observations, which on their own cannot establish causal relationships. Further exploration into causal impacts would require animal models and human trials to evaluate the interdependence between gut microbiota and nutrients in affecting VFM accumulation. Moreover, the use of self-reported dietary intake data is a clear limitation in our study as individuals tend to over report fruit and vegetable intakes, and under report food items that are considered to be unhealthy^50,51^. In addition to self-reported data, the time difference between FFQ data collection and gut microbiota profiling (on average 0.81 years) may not capture subtle temporary variations of the microbial community in response to food intake^52^. Furthermore, nutrient intake was corrected for total energy (kcal) intake to allow us to account for inadequate quantification of food intake, but this correction also removes the effect of overfeeding on VFM. Lastly, nutrient measurements used in this study do not account for their bioaccessibility and bioavailability that relies on the food matrix in which they are incorporated. Indeed, food microstructure and its processing greatly affect the bioaccessibility and bioavailability of nutrients^53^. Thus, to untangle the effect of nutrients on the host-microbiome crosstalk, future studies should also take into account the food source of the nutrients^54^. Finally, to confirm these results, similar analysis should be conducted in other cohorts including male subjects to increase generalizability.

To conclude, our study suggests that despite the importance of diet and nutrients in shaping gut microbiota composition, the gut microbes’ impact on VFM may not be nutrient dependent in many cases. However, the importance of nutrients in enhancing the growth of beneficial microbes and improving their effect on host health is not excluded. Moreover, we suggest that gut microbes may be necessary to trigger the effect of some (but not all) nutrients on the host, specifically biotin, vitamin E, fibre and magnesium. Although the synergistic effects of fibre, magnesium and the gut microbiota on host metabolism has been described, further investigation of the combined effect of gut microbes and vitamin E or biotin on VFM is also required. Finally, our work suggests that modulation of gut microbiota composition might be an effective target for VFM reduction in elderly females.

## Material and Methods

### Cohort

The sample included 1,760 female UK twins from the TwinsUK cohort. Briefly, the samples included Caucasian females of mean age 68 with available deep adiposity phenotype data, self-reported dietary intakes, and gut microbiota profiles^55^. The demographic characteristics of the study population are reported in Table 1. Ethical approval was granted by the National Research Ethics Service London- Westminster, the St Thomas’ Hospital Research Ethics Committee (EC04/015 and 07/H0802/84) and all experiments were performed in accordance with relevant guidelines and regulations. All research participants have signed the informed consent prior to taking part in the study.

### Food frequency questionnaire data and nutrient intake

Self-reported dietary intake was assessed by food frequency questionnaires (FFQ) following the EPIC-Norfolk guidelines^18^. Individuals answered questions on intake of 131 food groups and in case of multiple FFQ, only those at the nearest time point to faecal sampling for gut microbiota profiling were considered. On average the date of FFQ completion and date of fecal sampling differed by 0.81 years (SD = 1.77 years; Range: ±5 years). Nutrient intakes (weight/day) were derived from the UK Nutrient Database as previously described^6,56^. A total of forty-one nutrients were estimated from this calculation (Supplementary Table 1).

### Visceral fat mass

Visceral fat mass was measured by total body dual-energy X-ray absorptiometry (DXA) whole-body scans following manufacturer’s recommendations (QDR Discovery W system; Hologic, Inc., Bedford, MA). Participants were asked to lie flat and straight during the DXA procedure for the full body scan, as previously reported^17^. Visceral fat mass was calculated from one cross section of the whole body at the L4–L5 spinal segment (the two lowest vertebra of the lumbar spine), the typical location of a computed tomography (CT) slice, and is estimated in grams. It is defined as the mass of fat present in the abdominal cavity within the considered section. All scan printouts were reviewed by an expert reader to ensure proper positioning and analysis.

### Gut microbiota profiling

Gut microbiota composition was assessed *via* 16S rRNA gene sequencing, as previously described for the TwinsUK cohort^57^. Faecal samples were brought to the clinical visit or posted in sealed tubes with ice packs and then frozen at -80C on receipt by the lab. Samples were then shipped on dry ice to Cornell University where DNA was extracted and the V4 region of the 16S rRNA gene was amplified, and sequenced on the Illumina MiSeq platform. The data were quantified through estimation of OTUs. OTUs were generated from the 16S rRNA gene sequencing as previously described^58^. OTUs present in less than 25% of individuals were removed from the analysis. OTU counts were re-scaled to non-zero values, converted to relative abundances, and log10 transformed. Transformed abundances were corrected for technical covariates in a linear model including sequencing depth, sequence run, person who extracted the DNA, and person who loaded the DNA and sample collection method. The 582 OTU residuals from this model were used in all downstream analyses.

### Statistical analysis

Linear regression was carried out to assess tests of association using PopPAnTe^59^ a Java program that evaluates the association of quantitative data in related samples. In the pair-wise associations between VFM and gut microbiota composition (or VFM and diet) VFM was the response variable in the model and OTUs (or nutrients) were independent predictors along with covariates including family structure, age, BMI and average daily calorie intake (in kcal) estimated from FFQ. We adjusted for multiple testing using false discovery rate (FDR < 0.05)^60^.

To evaluate the effect of nutrients or OTUs on the VFM-OTUs or VFM-nutrients associations respectively, we conducted conditional analysis. These analyses repeat the pair-wise associations between VFM and OTUs (or VFM and nutrients), but now also taking into account diet (or gut microbiome composition) as main effects in the model. Conditional analyses were carried out for nutrients or OTUs significantly associated with VFM.

To formally assess the contribution of OTUs to the association between nutrients and VFM, we conducted mediation analysis using the “mediation” package in R^61^ where we considered OTUs as mediator for the causal effect of nutrients on VFM. Mediation analysis was performed using nutrients that were associated with both VFM and OTUs. As this package only supports the inclusion of one mediator, we performed a principal component analysis (PCA) on OTUs of interest and used the first principal component as the mediator. We used linear mixed effects models (lme4 package in R) for both the mediator and outcome models. For all models, we report the average causal mediation effect (ACME), the average direct effect (ADE) and the percentage of mediated effect. ACME represents the average size of the effect of a nutrient on VFM that is mediated by OTUs, while ADE represent the direct effect of nutrients on VFM. If both ACME and ADE are significant, the results suggest that the effect of a nutrient on VFM is partially mediated by OTUs. If only ADE is significant, OTUs are not involved in the association between nutrient and VFM. Finally, if only ACME is significant, then OTUs fully mediate the association between a nutrient and VFM.

To evaluate the relative importance of nutrients compared to gut microbiota composition as predictors for VFM, we performed random forest classification using the RandomForest package in R^20^. Classifier importance was recalculated using the same parameters as above with the gbm package in R^62^ using the Gaussian distribution, to evaluate the percentage contribution of each classifier to the model. A total of 982 individuals were selected by randomly taking one twin out of each pair to obtain a study group of unrelated individuals. This sample was then split into a training set (n = 700) and a test sample set (n = 282) using 500 trees. We performed one hundred permutations to evaluate the optimum number of variables to predict VFM.

A summary of the analysis conducted in this study can be found in Supplementary Fig. 3.

## Supplementary information

Supplementary information

Supplementary Dataset 1

Supplementary Dataset 2

Supplementary Dataset 3

Supplementary Dataset 4

Supplementary Dataset 5

## Data Availability

TwinsUK 16S rRNA gene sequencing data are available from the BioProject database under accession code PRJEB13747. Phenotype and diet data are available upon request through application to the TwinsUK data access committee. Information on data access and how to apply is available at http://twinsuk.ac.uk/resources-for-researchers/access-our-data/.
