# Supplementary material for: Dissecting the role of the gut microbiota and diet on visceral fat mass accumulation
Source: Sci Rep. 2019 Jul 5;9:9758. doi: 10.1038/s41598-019-46193-w (PMC6611773; doi:10.1038/s41598-019-46193-w)
Supplement: Supplementary file 1 — Supplementary information [file 41598_2019_46193_MOESM1_ESM.docx]

**Dissecting the role of the gut microbiota and diet on visceral fat mass accumulation**

Caroline I Le Roy^1^, Ruth E Bowyer^1^, Juan Castillo-Fernandez^1^, Tess Pallister^1^, Cristina Menni^1^, Claire Steves^1^, Sarah E Berry^2^, Tim D Spector^1^, Jordana T Bell^1^*.

^1^ The Department of Twin Research, Kings College London, 3-4th Floor South Wing Block D, St Thomas' Hospital, Westminster Bridge Road, SE1 7E

^2^ Department of Nutritional Sciences, King’s College London, Franklin-Wilkins Building, 150 Stamford Street, London SE1 9NH, UK

* correspondence to Jordana Bell: [jordana.bell@kcl.ac.uk](mailto:jordana.bell@kcl.ac.uk)

**Supplementary Information**

**
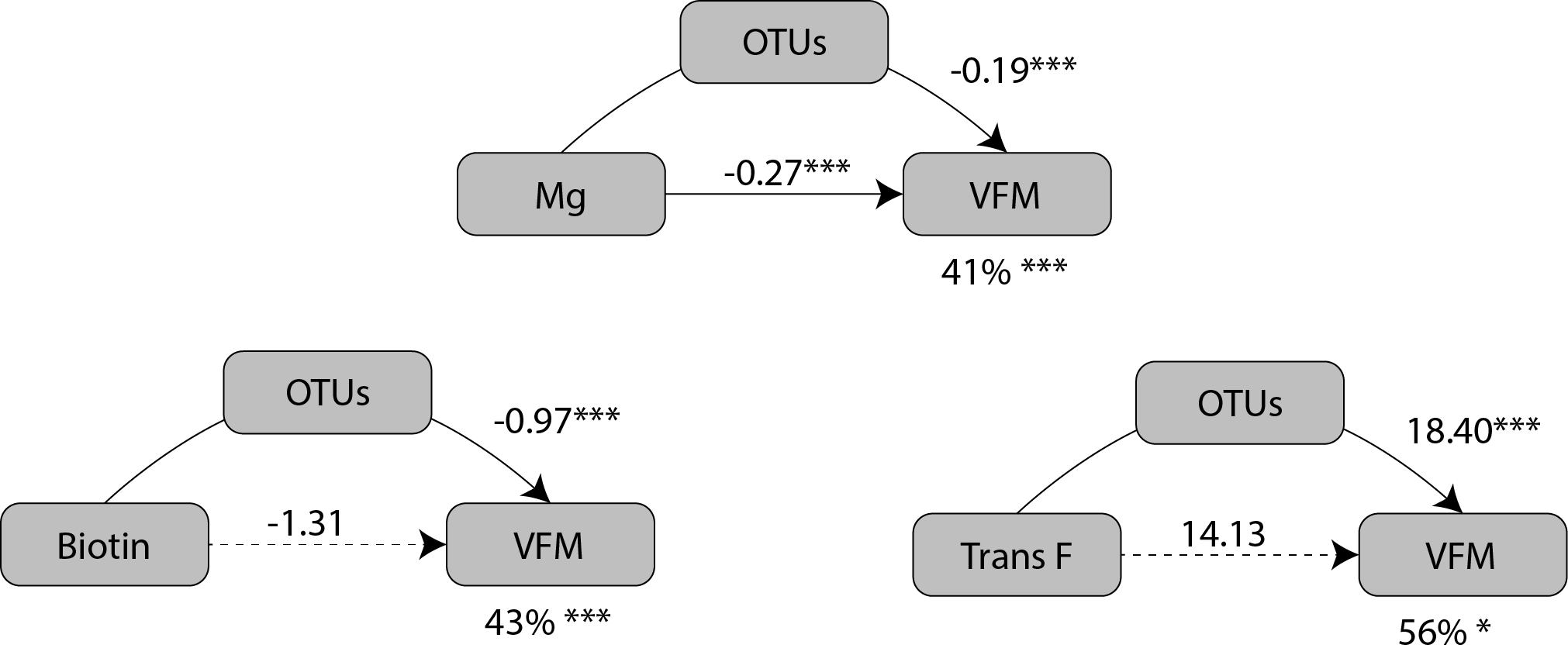
Supplementary figure 1**: Mediation of nutrients effect on VFM by OTUs. In each of the three models, the left box represents the causal variable (the nutrient), the top box the mediator (OTUs 1^st^ PC calculated based on the OTUs listed for each nutrient in **Supplementary table 6**) and the right box the response (VFM). The number by the top arrow represent the average causal mediation effect and the one on the bottom arrow the average direct effect. The number under the VFM box the percentage of mediation. * P < 0.05, ** P < 0.01, *** P < 0.001.

**
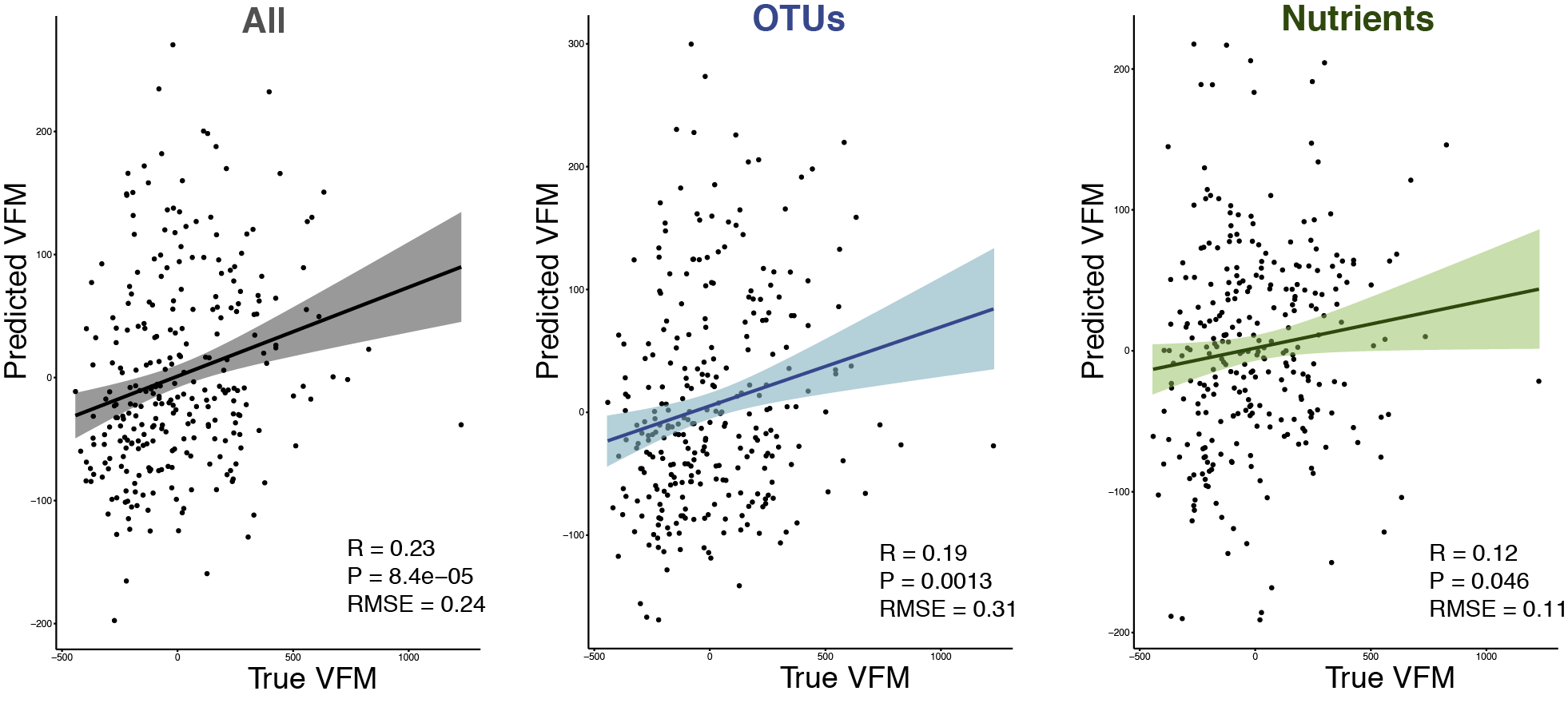
**

**Supplementary figure 2**: OTUs are better predictors of VFM than nutrients. Correlation between VFM and predicted VFM of the test set for RF models built using all 93 VFM-associated OTUs and 44 nutrients together or separately from left to right.


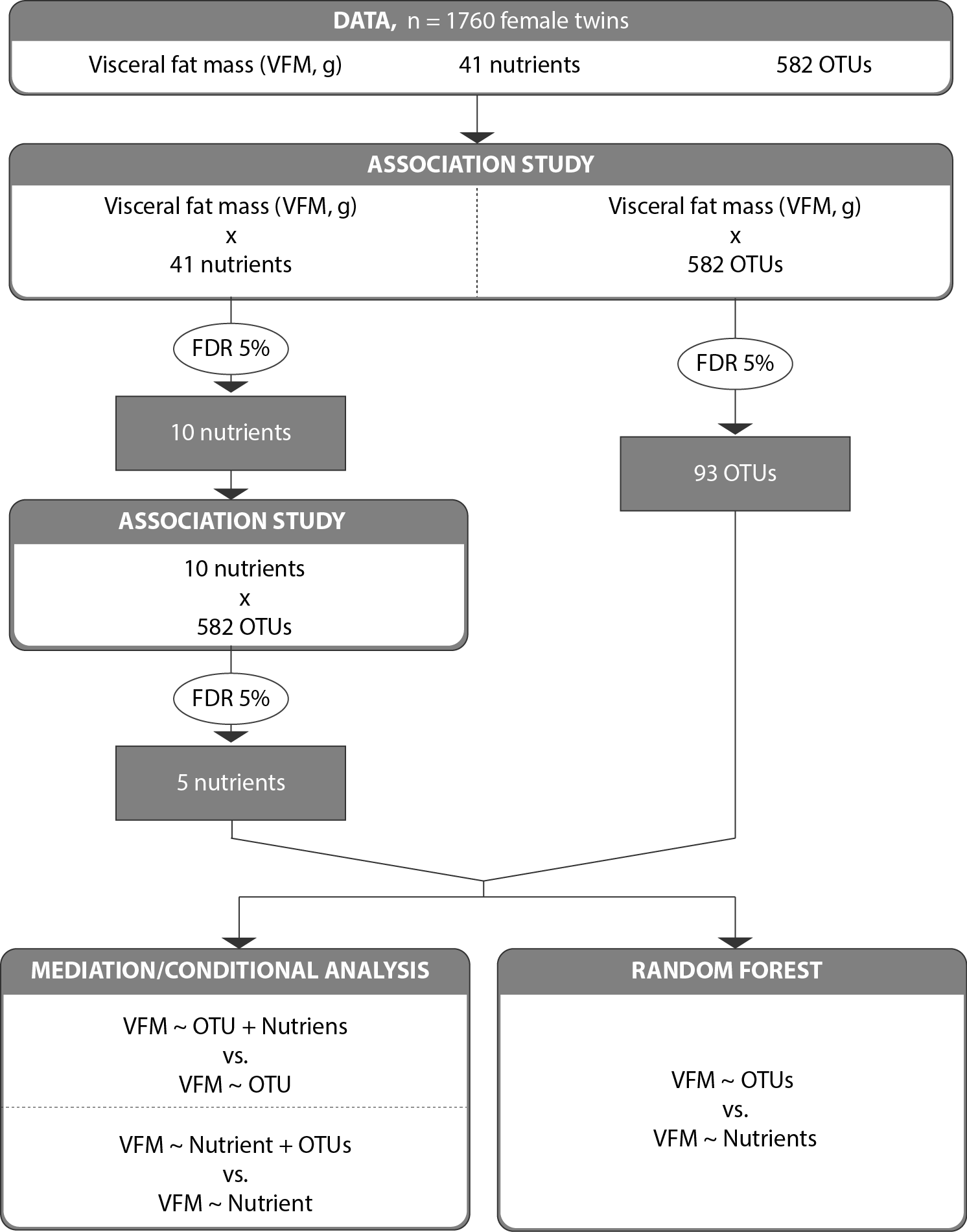


**Supplementary figure 3**: Flowchart of the study design.

**Supplementary table 1**: Summary statistics for the 44 nutrients used in this study

**Supplementary table 2**: Number of OTUs significantly (FDR < 5%) associated with each of the 44 nutrients.

**Supplementary table 3**: Summary of OUT-VFM associations that lose significance after correction for nutrients.

**Supplementary table 4**: Results of the association of OTUs with VFM following correction for nutrients.

**Supplementary table 5:** Description of OTUs used in the mediation analysis.
